# Diversity of Myxobacteria Isolated from Indonesian Mangroves and Their Potential for New Antimicrobial Sources

**DOI:** 10.1007/s00284-022-03066-2

**Published:** 2022-12-20

**Authors:** Senlie Octaviana, Gian Primahana, Tjandrawati Mozef, Luiz G. A. Borges, Dietmar H. Pieper, Joachim Wink

**Affiliations:** 1 Helmholtz Center for Infection Research, Microbial Strain Collection, Braunschweig, Germany; 2 Research Center for Applied Microbiology BRIN, Cibinong, Jawa Barat Indonesia; 3 Microbial Drug, Helmholtz Center for Infection Research, Braunschweig, Germany; 4 Research Center for Pharmaceutical Ingredients and Traditional Medicines BRIN, Cibinong, Jawa Barat Indonesia; 5 Microbial Interactions and Processes, Helmholtz Center for Infection Research, Braunschweig, Germany

## Abstract

Mangroves are unique intertidal ecosystems that provide ecological niches to different microbes, which play various roles in nutrient recycling and diverse environmental activities. The association between myxobacteria and mangroves are hitherto poorly understood. The aim of our study was to evaluate the myxobacterial community composition as well as isolate myxobacteria and to characterize the antimicrobial activity of myxobacteria isolates from Indonesian mangroves. Twenty-five cultivable myxobacteria were affiliated in six genera: *Myxococcus*, *Corallococcus*, *Archangium*, *Chondromyces*, *Racemicystis* and *Nannocystis* of the order *Myxococcales* based on partial 16S rRNA gene sequences. Thirteen crude extracts showed moderate activities against at least one of human pathogenic microorganisms. The crude extract of *Racemicystis* sp*.* strain 503MSO indicated a novel compound, which has not been reported in the database yet and the identification of this compound needs further study. The myxobacterial communities of three different sampling sites were analyzed using primers adapted for the myxobacteria group identification. The results showed that myxobacterial communities are more diverse than assumed. Therefore, our study has highlighted the importance of the mangrove habitat as promising harbor of myxobacteria as well as novel antimicrobial compounds with activity against pathogenic microorganisms.

**Supplementary Information:**

The online version contains supplementary material available at 10.1007/s00284-022-03066-2.

## Introduction

Mangroves are unique environments of small forest trees in brackish water with transitional zones in the coastal area. They tolerate a wide range of salinity, oxygen, nutrient and harbor many types of microorganisms [1, 2]. Different microorganisms origin from this habitat have been documented with different their bioactivity and compounds [3, 4]. However, there is a limited information about the antimicrobial potential of myxobacteria isolated especially from Indonesian mangroves, which are known as the world’s largest mangroves [5].

A preliminary study considered that marine environments are promising habitats for the isolation of antibiotic producing bacteria including myxobacteria. Myxobacteria are Gram-negative *Deltaproteobacteria* and it is one of the most fascinating groups producing natural microbial products [6, 7]. They produced more than a hundred new carbon skeletons and derivatives of compounds with antibacterial, antifungal, antimalarial, antioxidative, or antiviral activity [8–11]. Four genera of rare marine/saline origin are known so far and from two (*Haliangium* and *Enhygromyxa*) numerous (bioactive) secondary metabolites had been isolated like haliangicin, salimabromide, salimyxins, enhygrolides, and haliamide. Sharma & Thakur [13] noted that crude extracts with strong antimicrobial activities hint to the presence of promising antimicrobial compounds against infectious disease and treatment. Furthermore, these results were consistent with a previous study of Linares-Otoya et al. [12] on antimicrobial screening of predatory bacteria including myxobacteria from coastline. Therefore, it can be assumed that also saline environments like mangroves harbor an enormous potential of new antibiotic producing myxobacteria [14] and an exotic myxobacteria such as species *Racemicystis* for example from uncommon habitat should be explored.

Myxobacteria possess a social lifestyle with moving to get nutrients, preying to another microorganisms, and surviving from lack environment cooperatively in predatory groups [15]. Their communities are commonly found in various environments, even in extreme conditions [15–17]. A preliminary study considered the use of specific primers and probes to analyze myxobacterial communities in soil samples [18] in addition Li et al. [19] and Brinkhoff et al. [20] carried out PCR-DGGE (Denaturing Gradient Gel Electrophoresis) and quantitative real time PCR to define myxobacteria diversity. Clone bank analyses, cultivation approaches were also used to compare their diversity from different habitats [21]. Presently, next generation sequencing (NGS) of hypervariable regions of the 16S rRNA gene, is a tool that gives profound insights into microbial communities. Jiang et al. [22] stated tremendous success using this method in comparing the bacterial community diversity and composition between mudflat, edge, bulk, and rhizosphere of mangrove’s samples in Hong Kong, China. Furthermore, Linares-Otoya et al. [12] were able to detect predatory bacteria with NGS from the Peruvian coastline stating that the microbiome present in this region is a promising source for heterotrophic bacterial strains and has potential for bioprospecting of antibiotics. Nevertheless, there are no specific information about myxobacterial communities in mangrove habitat using the NGS method’s.

The use of culture-independent methods is of great interest, as it has identified a larger number of novel taxa when compared to culture-dependent methods under standard laboratory condition [12, 21–23]. However, the culture-dependent methods is important step to get novel antimicrobial compound, because without the successful isolated novel taxa from nature, the production of secondary metabolites never happened [14]. The aim of this study was to evaluate the myxobacterial community composition in Indonesian mangroves by Illumina sequencing of 16S rRNA genes using specific primers, to isolate myxobacteria, and to discover their potential for antimicrobial activity.

## Material and Methods

### Sample Collection

The mangrove samples were collected from Taman Muara Tawar, Bekasi (MT) (6°088772′N 107°002393′E), Taman Muara Angke, Jakarta (MA) (6°105321′N 106°735578′E), and Taman Mangrove Api-Api, Yogyakarta (MK) (7°894662′S 110°02554′E), at Java, Indonesia in November 2018 (Fig. S1). Total of six samples including sediment and leaf flakes were taken from each location. Approximately 50 g of the upper sediment with leaf, which fell on it, were placed in a sterile zip lock plastic bag. All samples were air dried at 30 °C for minimalizing contamination from fungi.

### Isolation of Myxobacteria

The strains were isolated as previously described by Mohr et al. [21] using water agar with autoclaved *Escherichia coli* strain K12 and Stan 21 agar with filter paper [24]. Swarming colonies or fruiting bodies were observed under dissecting microscope (Olympus SZX10) every 5–15 days and transferred to new water agar plates with *Escherichia coli* strain K12 and finally to VY/2 agar plates [25]. The pure cultures from VY/2 agar plates were transferred into 20 ml CY/H liquid medium [24] and 1.5 ml of well-grown cultures were directly conserved at −80 °C.

### Identification of Pure Cultures by 16S rRNA Gene Sequences

The bacterial DNA was extracted using Invitek Spin Plant Mini Kit (Invisorb) following the manufacturer’s instruction. One microliter of template DNA was directly amplified using F27/R1525 as described [21, 23]. The PCR products were checked by 0.8% agarose gel electrophoresis at 70 V for 40 min and purified by NucleoSpin Gel and PCR Clean up Kit (Macherey–Nagel, Düren, Germany). The forward and reverse sequences of 16S rRNA gene fragments were assembled with the BioEdit program [26] and closely related type strains were identified using the NCBI 16S rRNA gene database. Sequences were deposited at NCBI database under accession number MW199130, MW182265-MW182288.

### Microbiome Analysis

The total DNA was extracted from 250 mg of sample using the MOBio PowerSoil® Kit following the manufacturer’s instructions. The quality of DNA was measured in Nano-photometer IMPLAN. Amplification was performed using PrimerSTAR HS DNA Polymerase (Takara, Otsu, Shigu, Japan) following the manufacturer’s instructions. Amplification for analyzing the microbial community composition was described by Rath et al. [27] with modifications. Forward primers W2 and W5 [18] specifically targeting *Cystobacterineae* and *Sorangiineae*/*Nannocystineae* (Table S1) of the *Myxococcales* were separately used in conjunction with reverse primer R1525 [21, 23] in a first PCR reaction. Samples were pre-denaturated at 96 °C for 3 min following 25 cycles of denaturation at 94 °C for 1 min, annealing at 56.6 °C for 1 min, extension at 72 °C for 2 min. The PCR product was checked on 2.0% agarose gel electrophoresis and purified using NucleoSpin Gel PCR Clean up Kit (Macherey–Nagel, Düren, Germany). One microliter of purified PCR product was used as template in a second PCR with primers 807F and 1050R containing part of the sequencing primer sites as short overhangs (Table S1) for 20 cycles to enrich for target sequences. A third amplification step of 10 cycles added the two indices and Illumina adapters to amplicons [27]. The second and third PCR reaction following a first step PCR reaction. Obtained products were pooled in equimolar ratios and sequenced on an Illumina Miseq (2 × 300 bases, San Diego, USA).

The bioinformatic processing was performed as previously described [28]. Raw reads were merged with the Ribosomal Database Project (RDP) assembler [29]. Sequences were aligned within MOTHUR [30] (gotoh algorithm using the SILVA database [31] and subjected to preclustering (diffs = 2) yielding so-called phylotypes that were filtered for an average abundance of ≥ 0.001% and a sequence length ≥ 250 bp before analysis. The potential duplications of same *Myxococcales* sequences between both sets of primers pair were counted and were included to each primer pair analysis. Phylotypes were assigned to a taxonomic affiliation based on the naive Bayesian classification with a pseudo-bootstrap threshold of 80% [29, 32]. All sequences not matching to the order *Myxococcales* were deleted before further analysis. The relative abundance of genera was plotted using pivot table on Microsoft Excel. Sequences were deposited at NCBI database under accession number SRX9502207-SRX9502210 (Project PRJNA678217).

### Preparation for Crude Extracts

Seed cultures from the 25 isolates were prepared from myxobacterial swarming colonies after seven and before 15 days of incubation (depending on the myxobacteria growth rate) on VY/2 agar medium [24] by inoculation into 20 ml CY/H liquid medium [24]. The cultures were shaken at 160–180 rpm. After seven days, 10% of the seed cultures were transferred into 100 ml screening VY/2 liquid medium [24], which contained 2% XAD-absorber resin and incubated at room temperature for 7 up to 15 days. After 2% XAD-absorber was collected, eluate molecules bound on XAD was released using 70 ml using 70 ml acetone (J. T. Baker) and the organic phase was evaporated (Heidolph, Laborota 4003 control) at a temperature of 38–40 °C. The residue was mixed with 1 ml methanol (J. T. baker) and stored in a freezer at −20 °C.

### Screening for Antimicrobial Activity

All 25 crude extracts from 25 strains were evaluated by a test panel using the following pathogenic microorganisms: *Escherichia coli* WT-BW 25113, *Escherichia coli* JW0451-02, *Acinetobacteria baumanii* DSM 30008, *Citrobacter freundii* DSM 30039, *Pseudomonas aeruginosa* Pa14, *Staphylococcus aureus* Newmann, *Mycobacterium smegmatis* ATCC 700084, *Candida albicans* DSM 1665, *Wickerhamomyces anomalus* DSM 6766, and *Mucor hiemalis* DSM 2656. The antimicrobial activity was assessed by a growth inhibition test using a serial dilution of each crude extract against different pathogenic microorganisms in a 96-well plate [33]. The highest dilution, at which growth inhibition was observed, was noted. The antimicrobial activity was visualized quantitatively by heat map with Heatmapper software [34].

### Identification of Active Compound

The crude extract of *Racemicystis* sp. strain 503MSO was fractionated on 96-well plate to analyze the peak-activity correlation. Separation was conducted on an Agilent 1260 high performance liquid chromatography (HPLC) (Agilent Technology USA) using XBridge C_18_ column (Waters, Milford, Massachusetts, USA) (100 × 2.1 mm). The solvents consist of 5% acetonitrile (MeCN) in water (H_2_O) added with 5 mmol ammonium acetate (NH4OAc) and 0.04 ml/l acetic acid (CH3COOH) as solvent A and solvent B consist of 95% MeCN, 5 mmol (NH4OAc,) and 0.04 ml/l CH3COOH. Gradient system starting from 10% B to 100% B in 30 min and maintaining at 100% for 10 min, followed by post-run in 10 min for column re-conditioning. Flow rate was 0.3 ml/min and column temperature maintained at 40 °C. The fractions was collected every 30 s [35]. In paralel, the crude extract was analyzed by using diode array detector—high-resolution electrospray ionization mass-spectrometry (DAD-UV-HRESIMS) (MaXis ESI TOF, Bruker Daltonik GmbH, Bremen, Germany) using bridge ethylene hybrid (BEH) C_18_ column (Waters ACQUITY, Milford, MA, USA) (1.7 µm 2.1 × 50 mm). A linear gradient from 95% H_2_O and 5% MeCN to 5%H_2_O and 95% MeCN suplemented with 0.1% formic acid was used [33]. The eluate on 96-well plate was dried under streaming nitrogen (N_2_) gas and the respective bacteria was incubated directly to the plate. The clear zone on the well plate indicating the active compounds as well as the retention time in the respective mass chromatogram. The data were analyzed following Krug and Müller [36] and Hoffmann et al. [37] using Data Analysis 4.2 B383 (Bruker Daltonics) and identified with the in-house compounds library (Myxobase).

### Statistical Analysis

The results were analyzed using The IBM SPSS Statistics version 26.0 [38]. It was expressed as mean ± SEM (Standard Error of Mean). The differences between mean were statistically analyzed using one-way analysis of ANOVA with test of homogeneity of variances Bonferroni. *P* < 0.05 considered to statistical significance.

## Results

### Mangrove Myxobacteria Isolates

Seventy strains were successfully isolated from three different sources of Indonesian mangroves. All of the strains showed fruiting bodies formation and swarming on a surface agar medium. Therefore, based on different morphologies and 16S rRNA gene sequences analysis, the number of myxobacteria replicates from same source of organisms and location were reduced and 25 of 70 isolates were obtained. Twenty-five isolates, 16 from MA sampling site, 3 from MT sampling site and 6 from MK sampling site were selected for further analysis. Based on 16S rRNA gene sequence analysis, members of three suborders of *Myxococcales* were identified (see Table 1). Some isolates showed less than 98.60% similarity to close related type strains. Therefore, full genome sequencing is needed for their further characterization and whether they comprise novel species.

**Table 1:** Identities of myxobacterial isolates from Indonesian mangroves based on 16S rRNA gene sequences

No	Next related type strain	Type strain accession number	Sample name	Sample accession number	Similarity to type strain (%)	Sequence length (bp)	Sources	Location
	Suborder *Cystobacterineae*							
1	*Archangium gephyra* DSM2261^T^	DQ768106	455MSO	MW182273	98.07	883	Leaf flakes	MA
2	*Corallococcus coralloides* DSM2259^T^	NR074852	82MSO	MW182281	99.66	893	Soil	MA
3	*Corallococcus coralloides* DSM2259^T^	NR074852	101MSO	MW182265	99.22	896	Soil	MA
4	*Corallococcus coralloides* DSM2259^T^	NR074852	412MSO	MW182280	99.16	950	Leaf flakes	MT
5	*Myxococcus fulvus* DSM16525^T^	NR043946	35MSO	MW182276	98.33	897	Soil	MA
6	*Myxococcus fulvus* DSM16525^T^	NR043946	191MSO	MW182272	99.55	880	Soil	MA
7	*Myxococcus fulvus* DSM16525^T^	NR043946	411MSO	MW182283	99.36	932	Soil	MT
8	*Myxococcus fulvus* DSM16525^T^	NR043946	511MSO	MW182268	98.20	890	Leaf flakes	MK
9	*Myxococcus fulvus* DSM16525^T^	NR043946	483MSO	MW182269	99.33	891	Soil	MK
10	*Myxococcus macrosporus* DSM14697^T^	NR042331	471MSO	MW182285	99.88	860	Leaf flakes	MA
11	*Myxococcus macrosporus* DSM14697^T^	NR042331	161MSO	MW182282	99.78	896	Leaf flakes	MA
12	*Myxococcus macrosporus* DSM14697^T^	NR042331	451MSO	MW182288	99.34	916	Leaf flakes	MA
13	*Myxococcus macrosporus* DSM14697^T^	NR042331	21MSO	MW182275	98.71	928	Soil	MA
14	*Myxococcus macrosporus* DSM14697^T^	NR042331	173MSO	MW182271	98.76	885	Soil	MA
15	*Myxococcus macrosporus* DSM14697^T^	NR042331	421MSO	MW182284	99.70	928	Soil	MT
16	*Myxococcus macrosporus* DSM14697^T^	NR042331	521MSO	MW182286	99.10	893	Sandy Beach	MK
17	*Myxococcus macrosporus* DSM14697^T^	NR042331	431MSO	MW182287	99.78	929	Seaweed	MK
18	*Myxococcus macrosporus* DSM14697^T^	NR042331	532MSO	MW182267	97.66	992	Soil	MK
	Suborder *Sorangiineae*							
19	*Racemicystis crocea* DSM100773^T^	KT591707	503MSO	MW182266	98.30	890	Soil	MK
20	*Chondromyces robustus* DSM14608^T^	AJ233942	41MSO	MW199130	95.38	763	Leaf flakes	MA
21	*Chondromyces robustus* DSM14608^T^	AJ233943	151MSO	MW182279	95.34	1225	Leaf flakes	MA
22	*Chondromyces pediculatus* DSM14607^T^	GU207875	61MSO	MW182274	98.52	1012	Leaf flakes	MA
	Suborder *Nannocystineae*							
23	*Nannocystis pusilla* DSM53154^T^	NR117463	112MSO	MW182278	99.01	905	Soil	MA
24	*Nannocystis pusilla* DSM53154^T^	NR117463	182MSO	MW182270	99.56	899	Soil	MA
25	*Nannocystis pusilla* DSM53154^T^	NR117463	16MSO	MW182277	99.45	906	Leaf flakes	MA

MT: Bekasi, MA: Jakarta, MK: Yogyakarta

**Fig. 1:**
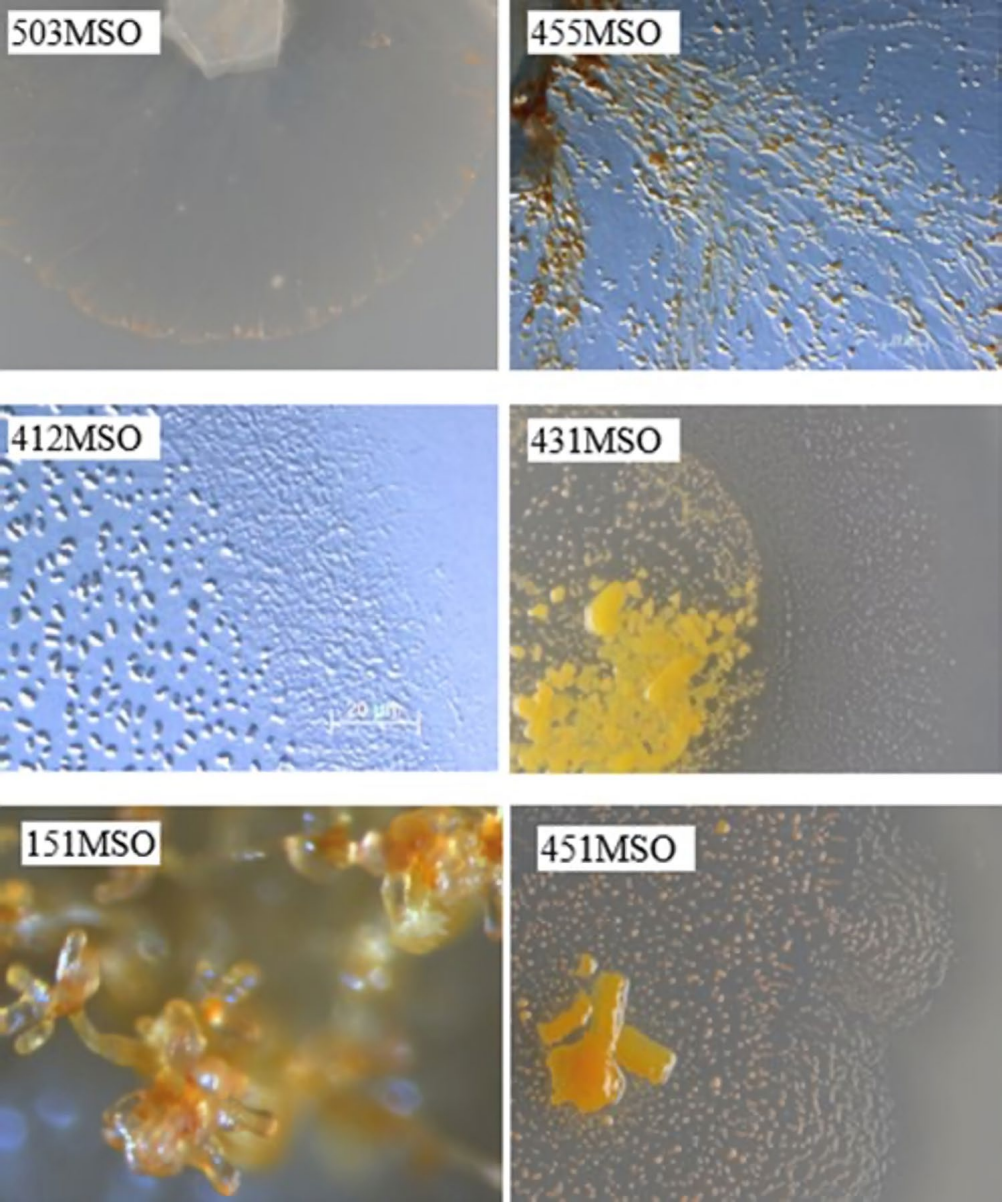
Myxobacteria isolated from Indonesia Mangrove: strain 503MSO with swarming area on VY/2 agar medium (**a**), fruiting bodies of strains 455MSO (**b**), 412MSO (**c**), 413MSO (**d**), 451MSO (**f**) on VY/2 agar medium, and strain 151MSO (**e**) on Stan21 agar medium

As main characteristics of myxobacteria as previously described by Reichenbach [16, 25], some of the swarming and fruiting bodies of myxobacterial isolates from mangroves can be seen in Fig. 1. *Myxococcus* sp. strains 431MSO and 451MSO have spherical fruiting bodies with yellow or oranges red colors on VY/2 agar medium. *Coralococcus* sp. strain 412MSO move with swarm colonies and forms fruiting bodies with coralloid-branched shapes. *Chondromyces* sp. strain 151MSO builds tree shaped fruiting bodies on Stan21 [39] agar medium and *Archangium* sp. strain 455MSO makes swarm colonies with branched radial veins on VY/2 agar medium. *Racemycistis* sp. strain 503MSO has swarming area like the genus *Sorangium* on VY/2 agar medium.

### Myxobacterial Diversity

Myxobacterial diversity was evaluated after amplification using two different primer pairs W2/R1525 and W5/R1525, targeting the suborders *Cystobacterineae* and *Sorangiineae/Nannocystineae*, respectively [18, 21, 23]. A total of 20,057 myxobacterial sequences (1761 ± 1002 Standard Error of Mean/SEM per sample) from 12 samples, where each sample was amplified twice with the two different primer pairs, were obtained. The success of the W2/R1525 primer to amplify members of the order *Myxococcales* order varied and between 0.56% and 70.42% of the obtained reads belonged to this order (see Table 2). With the W5/R1525 primer pair, 7.27–27.03% of amplified sequences were belonging to *Myxococcales*, indicating a low abundance of those bacteria in that sample. In contrast to the W5/R1525 primer pair, 70.42% of *Myxococcales* sequences with the W2/R1525 primer pair in one Yogyakarta mangrove sample indicate high abundance of *Myxococcales*. Moreover, sample MK from Yogyakarta contained more diverse myxobacteria than other and sample MT from Bekasi showed less diversity according to the results.

**Table 2:** The number of total reads sequences of *Myxococcales* in each sample

Samples	Total bacteria sequences in sample		*Myxococcales* sequences in sample		Percentage of *Myxococcales* in sample (%)		Location*
	W2/R1525	W5/R1525	W2/R1525	W5/R1525	W2/R1525	W5/R1525	
M39	8819	4816	437	350	4.96	7.27	MT
M41	21,064	10,537	315	674	1.50	6.40	MT
M44	12,053	3280	204	758	1.69	23.11	MA
M45	9991	6170	56	450	0.56	7.29	MA
M48	17,520	5428	12,338	1467	70.42	27.03	MK
M52	13,007	10,743	1446	1562	11.12	14.54	MK
Total	82,454	40,974	14,796	5261	–	–	
Mean	–	–	–	–	15.04	14.27	

*MT: Bekasi, MA: Jakarta, MK: Yogyakarta

**Fig. 2:**
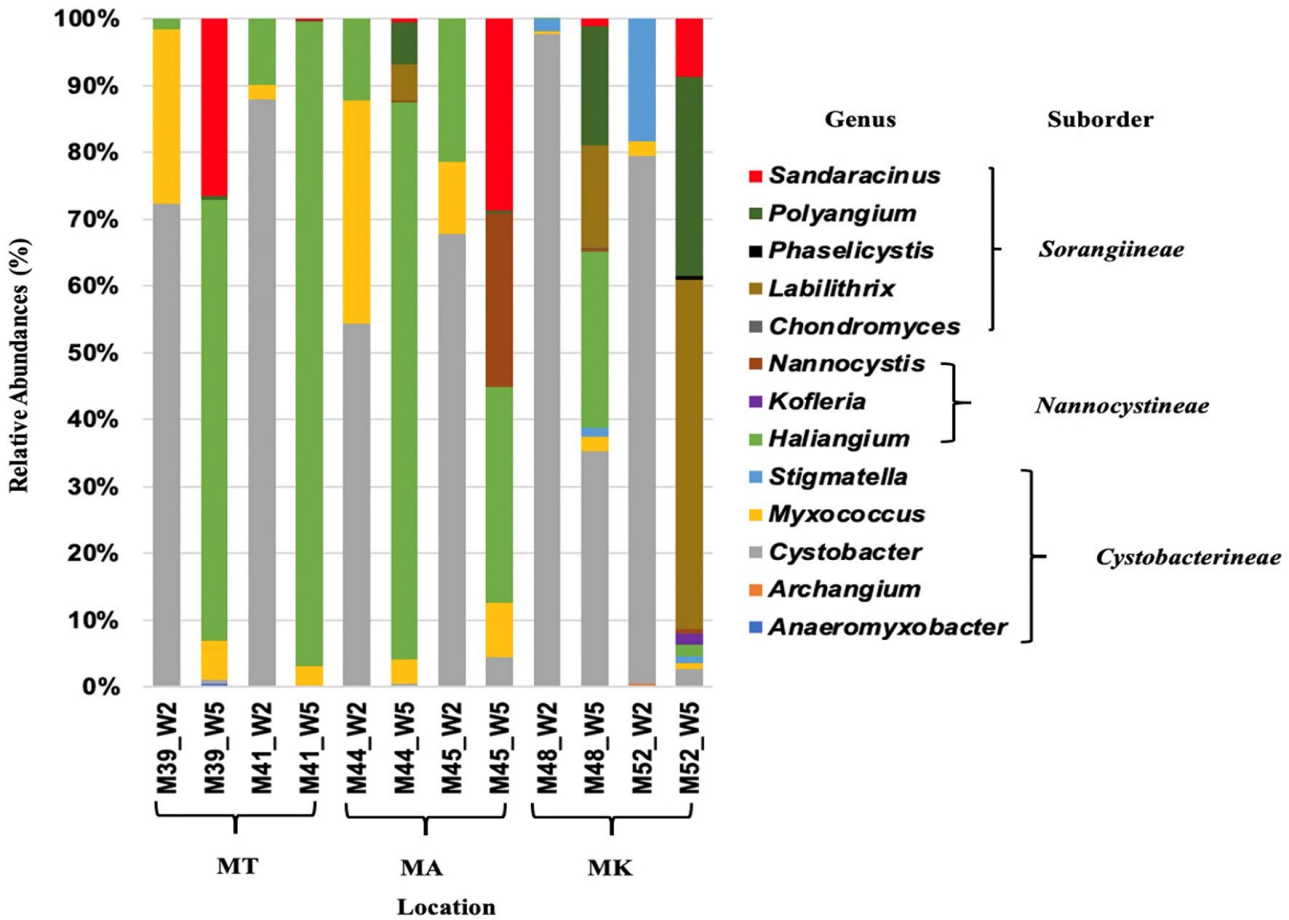
Relative abundances of myxobacterial genera in Indonesian Mangroves. MT: Bekasi MA: Jakarta MK: Yogyakarta using different set of primer W2/R1525 and W5/R1525. The community composition was revealed using primers targeting the suborder *Cystobacterineae* and the suborders *Sorangiineae/Nannocystineae*, respectively

Thirteen major genera were identified from three sampling sites analyzed in this study (Fig. 2). The W2/R1525 primer pair could also amplify sequences indicating the presence of *Cystobacter*, *Myxococcus, Stigmatella, Archangium* and *Anaeromyxobacter* of the *Cystobacterineae.* However, also *Haliangium* sequences was amplified by this primer. The W5/R1525 primer pair amplified sequence indicating the presence of members of the genera *Haliangium*, *Kloferia* and *Nannocystis* of the *Nannocystineae* and *Chondromyces*, *Labilithrix*, *Phaselicystis, Polyangium* and *Sandaracinus* of the *Sorangiineae.* In addition, some sequences of *Myxococcus, Stigmatella* and *Cystobacter* of the *Cystobacterineae* were observed as being amplified by this primer.

Overall, the primers showed good specificity for their targets. Clear differences in myxobacterial diversity could be observed in the sampling sites. *Stigmatella* spp. exclusively existed in MK samples, Yogyakarta mangroves whereas *Nannocystis* spp*.*, and *Labilithrix* spp*.* were present only in Jakarta and Yogyakarta mangroves. Only *Myxococcus* spp*.* were observed in all six samples, indicating that it is a common myxobacterium in mangroves samples (Table 3).

### Antimicrobial Activity of Myxobacteria

Twenty-five crude extracts from the 25 isolates were tested for their antimicrobial activity against ten pathogenic microorganisms, including five isolates Gram-negative bacteria, two-isolates Gram-positive bacteria and three fungi isolates (Table 3). Thirteen crude extract showed activity against at least one of the human pathogenic microorganisms tested.

**Table 3:** The statistical results of antimicrobial activity (MIC values µg/ml) of 25 crude extracts against 10 tested of microorganisms

Crude extract of strain	Tested of microorganisms									
	Gram-negative bacteria					Gram-positive bacteria		Fungi		
	*A. baumanii*	*E. coli* WT	*E. coli* acrB	*C. freundii*	*P. aeroginosa*	*M. smegmatis*	*S. aureus*	*M. hiemalis*	*W. anomalus*	*C. albicans*
455MSO	–	–	–	–	–	–	44.44 ± 19.23^j^	–	–	–
151MSO	–	–	–	–	–	–	55.57 ± 19.23^ k^	–	–	–
41MSO	–	–	–	–	–	–	55.57 ± 19.23^ k^	–	–	–
61MSO	–	–	–	–	–	–	55.57 ± 19.23^ k^	–	–	–
412MSO	–	–	–	–	–	–	55.57 ± 19.23^ k^	–	–	5.57 ± 2.40°
82MSO	–	–	–	–	–	–	55.57 ± 19.23^ k^	–	–	–
161MSO	–	–	11.11 ± 4.85^c^	66.67 ± 0^e^	–	–	44.44 ± 19.23^j^	–	–	–
35MSO	–	–	–	–	–	–	–	–	11.11 ± 4.82^ l^	22.22 ± 9.62^p^
421MSO	–	–	–	–	–	–	–	–	44.44 ± 19.25^ m^	–
431MSO	–	44.44 ± 19.25^a^	11.11 ± 4.85^c^	33.33 ± 0^e^	–	–	–	–	–	–
16MSO	–	22.22 ± 9.62^b^	10.89 ± 4.43^d^	22.22 ± 9.61^f^	–	–	44.44 ± 19.23^j^	–	–	–
503MSO	–	–	–	–	–	–	55.57 ± 19.23^ k^	–	55.55 ± 19.25^n^	44.44 ± 19.24^q^

The superscript alphabet indicated statistically significant difference at the 0.05 level

The crude extract of 101MSO, 173MSO, 191MSO, 21MSO, 411MSO, 451MSO, 471MSO, 483MSO, 511MSO, 521MSO, 532MSO, 112MSO and 182MSO did not exhibit antimicrobial activity

Two of them showed activity against both Gram-negative strains *Escherichia coli* and *Citrobacter freundii* and nine were active against Gram-positive *Staphylococcus aureus.* Furthermore, three of the extracts showed activity against the yeasts *Candida albicans* and *Wickerhamomyces anomalus.*

The crude extract of *Racemicystis* sp. strain 503MSO was fractionated in order to identify the responsible active compounds. We considered and selected *Racemicystis* sp. strain 503MSO out of 13 active strains because of ≤ 98.60% 16S rRNA sequence similarity to the type strain and because of missing information about compounds from genus *Racemicystis* in the Myxobase database.

The crude extract of *Racemicystis* sp. strain 503MSO was fractionated in order to identify the responsible active compounds. The compound identification was done by comparing detected mass of the parent ions of the active fractions with in-house Myxobase database. The Myxobase is a database to support research with myxobacteria, which are increasingly recognized as producers of secondary metabolites. Within Myxobase, the information of bioactivity, retention time, UV spectrum, molecular mass and elemental formula of the molecule responsible for the active peak and HPLC chromatogram were provided. A summary of the active compounds from strain 503MSO was provided in Figure S1.

Two active fractions from *Racemicystis* sp*.* strain 503MSO were identified comprising compounds with masses of *m/z* 375.2531 and 604.3857 [M + H]^+^, respectively after the high-resolution mass-spectrometry (HR-MS) analysis of these active fractions. The peak chromatogram which indicated the presence of compound from the crude extract was mentioned in Fig. S1.

## Discussion

Due to the increasing need for new antibiotics, the present study focused on the evaluation of the myxobacterial community as well as the isolation of novel members of the order *Myxococcales* and their potential for antimicrobial activity. Recent isolations of members of the myxobacteria have frequently resulted in the identification of new secondary metabolites with antibiotic activities. Untapped habitats such as various marine ecosystem have been proven to be a valuable resources for new microorganisms [7, 40, 41]. Indonesian mangroves explored in the present study were unexploited marine habitats and have a huge potential for the isolation of new uncommon species of myxobacteria.

Twenty-five strains were isolated from three different sites of Indonesian mangroves by conventional methods for isolation. *Myxococcus* strains were presented in all six samples and it reflected both myxobacteria isolates versus myxobacterial communities that were amplified based on 16S rRNA gene sequences. According to the fruiting body formation of *Myxococcus*, they were easy to be purified and cultivated under laboratory conditions [42]. Previously, this genus was used as model for wide-ranging studies of biotechnology [15, 43–46].

The analysis of myxobacterial communities by culture-independent methods revealed more abundances than myxobacterial isolates by culture-dependent method. For instance, the culture-independent community analyses revealed the genus Haliangium to be one of the most dominant and common myxobacteria in MA and MT samples, whereas it was not found by the culture-dependent method in our study. The faster growing fungal or bacterial contaminants exhaust nutrients before slow growing myxobacteria could be observed and further, the insufficient growth conditions in laboratory missing requirements such as changing tidal-caused salinity might be reasons that rare myxobacterial species were not isolated [21].

Thirteen out of 25 crude extracts from myxobacterial isolates yielded different activities against pathogenic microorganisms. These predatory bacteria including myxobacteria from coastline may be promising sources for novel antibiotics [12].

In this study, we only focused on the active crude extract of *Racemicystis* sp*.* strain 503MSO, an isolate from Yogyakarta mangroves. *Racemicystis* sp*.* strain 503MSO was selected for further analysis because there was no information about potential bioactive compounds from the genus *Racemicystis* in the Myxobase database yet. Therefore, it has a higher possibility to get new compounds from this genus.

The active fraction of *Racemicystis* sp. strain 503MSO with *m/z* 375.2531 [M + H]^+^ and 604.3847 [M + H]^+^ were detected in this study and may represent so far, an undescribed compound. Therefore, *Racemicystis* sp. strain 503MSO should be further explored including substantial work for optimizing isolation, structure elucidation and biological activity of the pure substance. To the best of our knowledge no bioactive compounds from the same genus and other sources were reported in the scientific publication to date.

Indonesian mangroves were inhabited by complex myxobacterial communities as shown by amplification using primer targeting *Cystobacterinae* (W2/R1525) and *Sorangiineae/Nannocystineae* (W5/R1525) [18, 21, 23]. Previously, Linares-Otoya [12] observed that the use of universal primers (F515/R806) yielded only a neglectable amount of myxobacterial sequences (equivalent to 0.0002%) as also indicated by our work.

The rapid sequencing-based analysis of myxobacterial communities has advantages compared to previous studies comprising DGGE (Denaturing Gel Gradient Electrophoresis) [47], hybridization analysis of a 16S rRNA gene library [18] or library analysis [21, 23] and by use of two PCR reactions an overview of all three suborders, i.e., *Cystobacterineae*, *Sorangiineae* and *Nannocystineae* were obtained [48, 49]. However, with the current method, a high amount of non-*Myxococcales* genera were amplified and target sequences often comprised < 10% of sequences. Evidently, optimization of primers is still necessary. Moreover, a species level information is only possible in exceptional cases using the V5V6 hypervariable region as performed here.

Overall, mangrove habitats are a rich source for antimicrobial compounds produced by myxobacteria, highlighting the necessity to explore their diversity.

## Conclusions

This study confirmed that neglected areas such as mangroves are promising habitats for the isolation of novel myxobacteria strains and potential sources for unknown secondary metabolites producers. Based on comparison of its monoisotopic mass and retention time of compound described in Myxobase database, we suggest further studies for isolating and upscaling active compound of *Racemicystis* sp. strain 503MSO and applying novel models of biological test as well.

## Supplementary Information

Below is the link to the electronic supplementary material.

Supplementary file1 (TIFF 111 KB)

Supplementary file2 (TIFF 1804 KB)

Supplementary file3 (DOCX 14 KB)

## Data Availability

The data presented in this study are available in the Supplementary Information and at NCBI database under accession number SRX9502207-SRX9502210 (Project PRJNA678217).

## Code Availability

The authors declared that the consent for code availability is not applicable.
