# Supplementary figures and images for: Diversity of Myxobacteria Isolated from Indonesian Mangroves and Their Potential for New Antimicrobial Sources
Source: Curr Microbiol. 2022 Dec 20;80(1):46. doi: 10.1007/s00284-022-03066-2 (PMC9768008; doi:10.1007/s00284-022-03066-2)

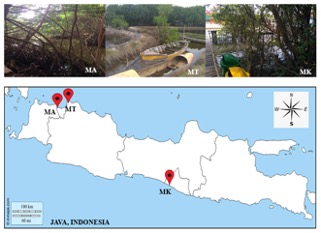

Supplement: Supplementary file 1 — Supplementary file1 (TIFF 111 KB) [file 284_2022_3066_MOESM1_ESM.tiff]

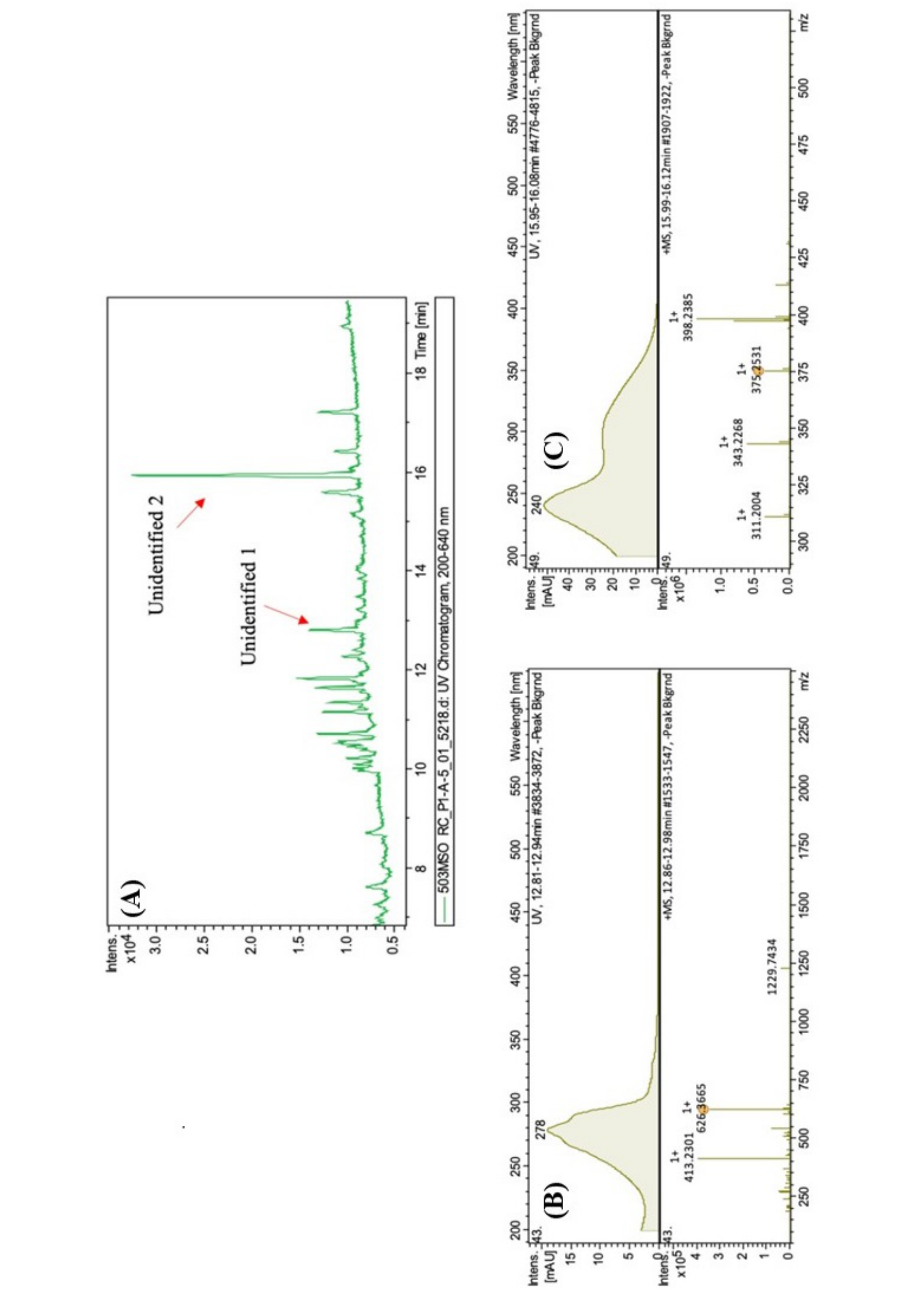

Supplement: Supplementary file 2 — Supplementary file2 (TIFF 1804 KB) [file 284_2022_3066_MOESM2_ESM.tiff]
