# Supplementary material for: Diversity of Myxobacteria Isolated from Indonesian Mangroves and Their Potential for New Antimicrobial Sources
Source: Curr Microbiol. 2022 Dec 20;80(1):46. doi: 10.1007/s00284-022-03066-2 (PMC9768008; doi:10.1007/s00284-022-03066-2)
Supplement: Supplementary file 3 — Supplementary file3 (DOCX 14 KB) [file 284_2022_3066_MOESM3_ESM.docx]

ORIGINAL RESEARCH

Diversity of Myxobacteria Isolated from Indonesian Mangroves and Their Potential for New Antimicrobial Sources

Senlie Octaviana ^1, 2^, Gian Primahana ^3, 4^, Tjandrawati Mozef ^4^, Luiz G. A. Borges ^5^, Dietmar H. Pieper ^5^, and Joachim Wink ^1,^ *

^1^ Microbial Strain Collection, Helmholtz Center for Infection Research, Braunschweig, Germany

^2^ Research Center for Applied Microbiology BRIN, Cibinong, Jawa Barat, Indonesia

^3^ Microbial Drug, Helmholtz Center for Infection Research, Braunschweig, Germany

^4^ Organization Research for Health, National Research and Innovation Agency BRIN, Cibinong, Jawa Barat, Indonesia

^5^ Microbial Interactions and Processes, Helmholtz Center for Infection Research, Braunschweig, Germany

***** Correspondence

Joachim Wink, Email : Joachim.Wink@helmholtz-hzi.de

**SUPPORTING INFORMATION**

**Table S1**. List of primer in this study

| **Primer** | **Sequence** |
| --- | --- |
| 807F with overhang | ACGACGCTCTTCCGATCT**GGATTAGATACCCBRGTAGTC** |
| 1050R with overhang | GACGTGTGCTCTTCCGATCT**AGYTGDCGACRRCCRTGCA** |
| W2 | GTAAAGCACTTTCGACCG |
| W5 | GTAAGACAGAGGGTGCAAACGT |
